# Microbiological profile and infection potential of different cryopreserved skull flaps after decompressive hemicraniectomy. Is cryopreservation at − 80 ℃ better?

**DOI:** 10.1186/s13104-022-06042-y

**Published:** 2022-05-13

**Authors:** R. Agrawal, C. Rompf, A. B. Pranada, P. Vollmar, A. De Lorenzo, A. Hoyer, K. Gousias

**Affiliations:** 1 Department of Neurosurgery, St Marien Academic Hospital Luenen, University of Muenster, KLW St. Paulus Corporation, Altstadtstrasse 23, 44532 Luenen, Germany; 2 Medical School, Rheinische Friedrich-Wilhelms University of Bonn, Venusberg-Campus 1, 53127 Bonn, Germany; 3 Department of Medical Microbiology, MVZ Dr. Eberhard & Partner Dortmund, Balkenstrasse 17-19, 44137 Dortmund, Germany; 4 Department of Psychiatry, LVR, University of Essen-Duisburg, Duisburg, Germany; 5 Biostatistics and Medical Biometry, Medical School OWL, Bielefeld University, Universitätsstrasse 25, 33615 Bielefeld, Germany; 6 Medical School, Westfaelische Wilhelms University of Muenster, Albert-Schweitzer-Campus 1, 48149 Muenster, Germany; 7 Medical School, University of Nicosia, 2408 Nicosia, Cyprus

**Keywords:** Decompressive hemicraniectomy, Skull bone flaps, Cryostorage, Skull infection

## Abstract

**Objective:**

Patterns of cryopreservation of explanted skull bone flaps have long been a matter of debate, in particular the appropriate temperature of storage. To the best of our knowledge no study to date has compared the microbiological profile and the infection potential of skull bone flaps cryostored at the same institution at disparate degrees for neurosurgical purposes. In the context of our clinical trial DRKS00023283, we performed a bacterial culture of explanted skull bone flaps, which were cryopreserved lege artis at a temperature of either − 23 °C or − 80 °C after a decompressive hemicraniectomy. In a further step, we contaminated the bone fragments in a s uspension with specific pathogens (*S. aureus, S. epidermidis and C. acnes*, Colony forming unit CFU 10^3^/ml) over 24 h and conducted a second culture.

**Results:**

A total of 17 cryopreserved skull flaps (8: − 23 °C; 9: − 80 °C) explanted during decompressive hemicraniectomies performed between 2019 and 2020 as well as 2 computer-aided-designed skulls (1 vancomycin-soaked) were analyzed. Median duration of cryopreservation was 10.5 months (2–17 months). No microorganisms were detected at the normal bacterial culture. After active contamination of our skull flaps, all samples showed similar bacterial growth of above-mentioned pathogens; thus, our study did not reveal an influence of the storage temperature upon infectious dynamic of the skulls.

## Introduction

The decompressive hemicraniectomy (DC), i.e. the removal of part of the skullcap, is an established surgical method to manage life-threatening increased intracranial pressure as a consequence of a malignant brain swelling [1–3]. The latter may arise in the context of a severe traumatic brain injury (sTBI) or a major cerebral infarction. A DC is then urgently indicated, since the contrary preservation of an intact rigid skull bony shell is highly associated with a critical entrapment of the brain stem into the tentorium slit. To this end, large prospective studies have confirmed a reduced mortality in patients, who underwent a DC after a malignant cerebral infarction compared to conservative managed patients [4–6].

The reconstruction of the DC-related cranial defects, called cranioplasty, is performed after the subsidence of the brain swelling and may be conducted either via reimplantation of their autologous explanted skull flap or implantation of a synthetic computer-aided-designed (CAD) bone flap. Currently, there is no standard method of handling the explanted skull flaps. A traditional method is to preserve the skull flaps in a subcutaneous pocket in the abdominal wall [7, 8]. Alternatively, the bone flap may be stored in a medical freezer at – 23 ℃ to – 80 ℃ using aseptic technology [9, 10].

Although cranioplasty may be characterized as one of the simplest neurosurgical interventions, this procedure is related to a high risk of postoperative complications [11–13]. The complication rate of infections and autolysis of the reimplanted skull flaps is as high as 7–22% and 3–51%, respectively [3, 14, 15].

In order to analyze different patterns of cryostorage as a potential prognostic factor for later infections of skull flap implants, we conducted a retrospective study comparing the microbiological profile and the infection potential of explanted skull flaps stored at − 23 °C vs. − 80 °C.

## Main text

### *Material* and methods

#### Patients

The clinical trial DRKS00023283 (http://apps.who.int/trialsearch/) aimed to clarify whether different patterns of storage of explanted skull flaps after DC, in particular storage at different degrees of  ℃, may prognosticate late complications after cranioplasty, among others infection of the skull flaps implants. To this end, we analyzed explanted skull flaps of only deceased adult patients, who underwent a DC in our Department between June 2019 and October 2020 for an acute malignant brain swelling. The trial has been approved by the local ethic committee of University of Muenster (ethic votum: 2020-340-f-S). Informed consent for inclusion in the research and publication was obtained by their legal representatives.

**Fig. 1:**
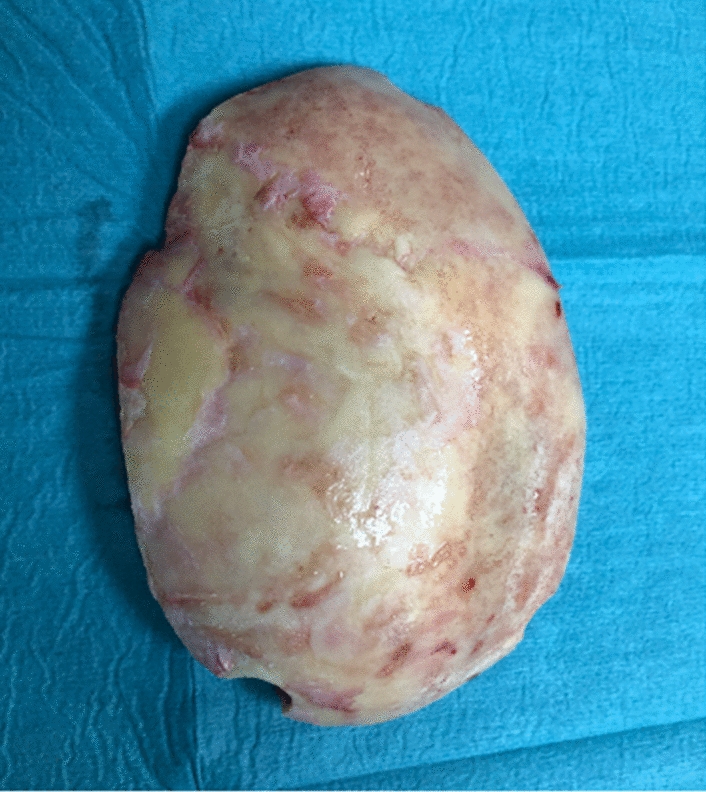
Skull flaps explanted after DC used for bacterial culture

**Fig. 2:**
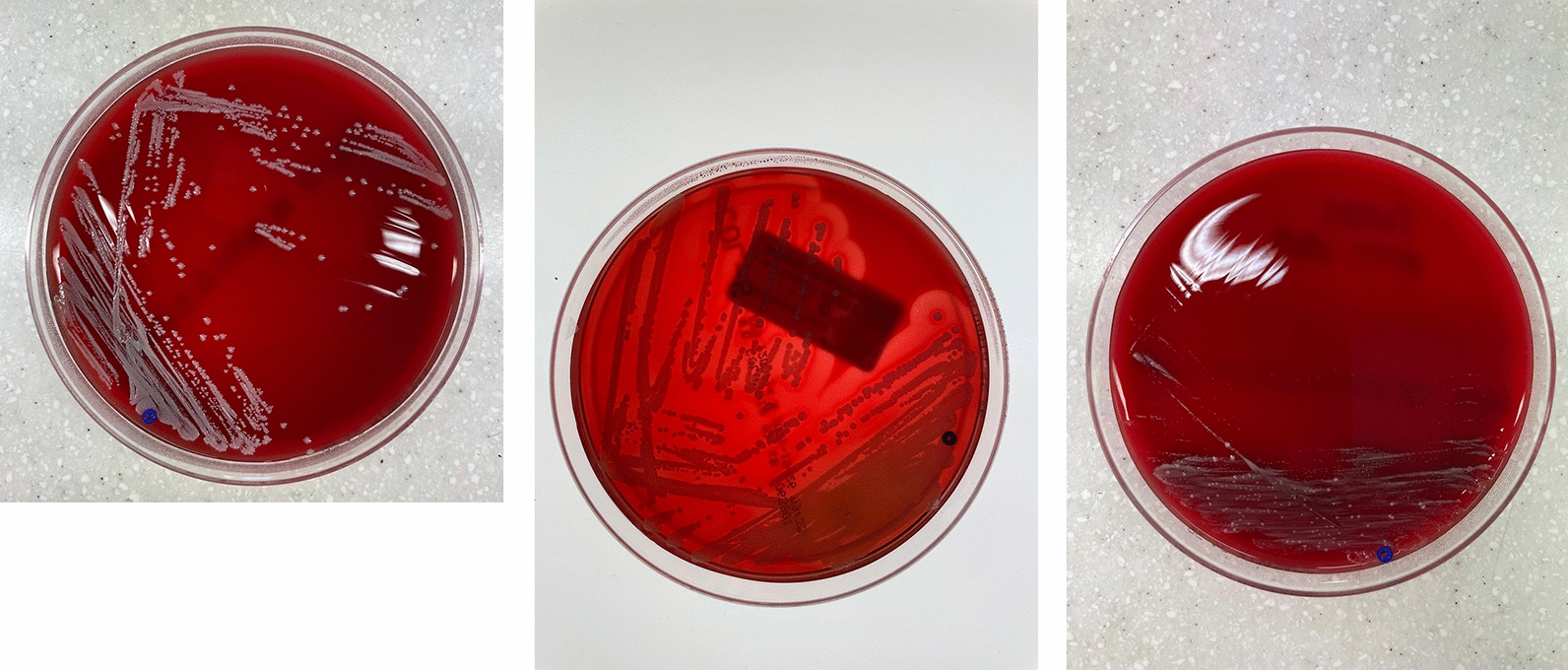
Typical bacterial growth after contamination with S. aureus, S. epidermidis and C. acnes

We included in our assays explanted skull flaps of 17 patients, 8 skulls were preserved at − 23 °C (group A), whereas 9 at − 80 °C. In addition, we assessed another group C, which comprised 2 sterile CAD (1 vancomycin-soaked). Specific parameters of demographics, surgical procedure and storage, i.e. age (years), sex (m/w), cause of malignant brain swelling (stroke vs. sTBI), additional skull fracture (yes/no), infection prior to DC (yes/no), duration of DC (minutes), duration (months) and temperature of cryostorage (23 °C vs. − 80 °C) have been included as potential prognostic factors in our statistical analysis (Fig. 1). As endpoints of our study were defined (1) the identification of microorganisms in the bacterial cultures (yes/no) and (2) in case of growth, the patterns of the colonies (Fig. 2) of microorganisms, f.i. concentration of microorganism.

### Material collection, storage and microbiological analysis

The bone flaps were collected during DC, sterile packed in triple plastic bags and stored in a medical freezer at a temperature of either − 23 °C (DCs between June and November 2019) or − 80 °C (DCs between December 2019 and October 2020). For the purposes of the microbiological assays, the skulls were thawed at room temperature for 2 h using strictly aseptic technology. The bone flaps were then crushed with a hammer and bone rongeur forceps; five of the resulting centrally located bone fragments (cortex and cancellous bone, sized approximately 0.8 × 2 cm) of each skull flap, have been processed for further microbiological investigations. Two of these fragments were used for aerobic and anaerobic cultures, respectively, according to quality standards of microbiological diagnostic [16].

For the purposes of the aerobic microbiological analysis each bone fragment was rolled out onto chocolate agar (Chocolate PolyViteX Agar, bioMérieux, Marcy l’Étoile, France) and blood agar (Columbia agar with 5% sheep blood, BD, Heidelberg) and at least put in liquid brain heart infusion (BBL™ Brain Heart Infusion, BD, Heidelberg) for enrichment. By analogy, anaerobic culture was performed with an anaerobic blood agar plate (Schaedler agar with 5% sheep blood, BD, Heidelberg) and liquid thioglycolate medium (BBL™ Enriched Thioglycollate Medium with vitamin K1 & Hemin, BD, Heidelberg) for enrichment. The inoculated media were incubated for 14 days at 35 °C ambient air plus 5% CO_2_. All media were assessed for growth at 48 h, 7 and 14 days. In case of positive cultures, extent of growth was quantified in a semiquantitative manner by categorizing as light, moderate or heavy growth. If solid media showed no growth after 14 days of incubation, subcultures from the liquid enrichment media were performed on blood agar or anaerobic blood agar, respectively, followed by an incubation period of three days under conditions described above. MALDI-TOF MS was employed for bacterial identification.

In a second step, contamination experiments were performed with the remaining three of the five fragments of each skull flap (group A and B) as well as the three fragments of each of the CAD skulls (group C). Each of the bone and CAD fragments were transferred to a suspension of the reference strains *Staphylococcus aureus* ATCC 25923, *Staphylococcus epidermidis* ATCC12228, and *Cutibacterium acnes* ATCC 6919 at a concentration of 10^3^ colony forming unit (CFU)/ml in PBS and then stored in a refrigerator overnight at 5 °C.

The following morning, the contaminated fragments were wiped dry on a sterile gauze paid. Subsequently, *S. aureus*- and *S. epidermidis*-contaminated fragments were rolled out onto chocolate blood agar plates, whereas *C. acnes*-contaminated fragment onto anaerobic blood agar plate. Each fragment was then placed in thioglycolate medium. Culture conditions were chosen and procedures were conducted as described above.

Standard statistical methods were used for the comparisons between the subgroup. Additionally, we used a linear mixed model to account for repeated measurements. P-values < 0.05 were considered as statistically significant.

## Results

A total of 17 cryopreserved skull flaps (8 at − 23 °C; 9 at − 80 °C) obtained during DC between June 2019 and October 2020 as well as 2 CAD (1 vancomycin-soaked) were analyzed (Table 1). Median age of our cohort was 70 years, whereas 9 patients (53%) were male. 6 patients underwent a DC after severe TBI; 11 patients for a vascular disease like infarct or spontaneous bleeding. Median duration of DC was estimated at 125 min, no infections prior to DC were noticed, 17.6% of the skulls were fractured. Median duration of cryopreservation was 10.5 months (2–17 months).

**Table 1:** Study group demographics

Variable	− 23 °C	− 80 °C	P value
N	8	9	
Age (median, Q1; Q3)	67.5 (58; 77.5)	71 (53; 76)	n.s
Males	4 (50%)	5 (55.5%)	n.s
Traumatic Brain injury	2 (25%)	4 (44.5%)	n.s
Skull fracture	1 (12.5%)	2 (22.2%)	n.s
Preoperative infections prior to DC	0 (0.0%)	0 (0.0%)	n.s
Duration of surgery (median, Q1, Q3)	129.5 (122; 210)	125 (120; 160)	n.s
Postoperative infections after DC	4 (50%)	4 (44.5%)	n.s

No significant differences in the study group demographics (Group A: − 23 °C vs. B: − 80 °C) have been noticed. However, skull flaps were stored for a significant longer period at − 23 °C vs. − 80 °C (13.5 months vs. 7 months, p = 0.003)

No microorganisms were detected at the normal bacterial culture. After active contamination of our skull flaps (A, B and C), all samples showed similar bacterial growth curve of above-mentioned pathogens.

## Discussion

Despite the surgical simplicity of cranioplasty, this procedure is related to an unusually high rate of early and late complications up to 51% [11–13]. One of the most common complication is secondary bone infection (7–22%) [3, 14, 15]. Skull bone infections are still observed, even in the case of additional sterilization or various antimicrobiological methods prior to reimplantations of the explanted cryostored skull flaps, like autoclaving. Fan et al. cryopreserved 989 bone flaps in liquid Nitrogen, thus − 196 °C, using dimethyl sulfoxide as a cryoprotectant, and still found an infection rate of 4.06% after cranioplasties. Wui et al. sterilized the at − 70 °C preserved explanted skull flaps additionally by autoclaving before reimplantation; nevertheless, an infection rate of 38.5% was documented [17]. Tahir et al. placed the at – 26 ℃ cryopreserved autologous bone flaps in a solution of normal saline, hydrogen peroxide and povidone iodine solution mixed with antibiotics before reimplantation but still 3.4% of the patients showed postoperative infections [18].

To date, several factors have been identified to associate with a higher probability of postoperative bone infection, namely younger age, reimplantation of multi-fragmented skull flaps, a ventriculoperitoneal shunt mandatory hydrocephalus, multiple skull operations or long cryopreservation of the explanted skull flaps [9, 19–22].

A suboptimal temperature during storage of the bone flap may also be speculated as a cause of increased risk for postoperative infections. Since large prospective clinical trials allow cryostorage of patients’ material only at temperatures equally or lower than − 80 ℃ in order to limit the cell metabolic activities and therefore cell damage, some may also anticipate lower infection rates of reimplanted skull flaps, which have been preserved at − 80 ℃ rather than – 23 ℃. Given the paucity of relevant prospective studies, only retrospective studies on patients’ series are available to handle this theme. However, also these studies are not eligible to investigate for the optimal temperature of storage, since no statistically powerful comparisons between series treated at different institutes with disparate patterns of skull flaps storage are feasible due to the selection and treatment bias. Since no reasonable comparisons between series of different institutes can be made, the current literature simply documents postoperative infections after cranioplasties regardless of the chosen temperature of storage [23, 24]

A different approach to give more insights into the role of skull flaps storage in the risk of postoperative skull bone infections would be a direct analysis of the skull flaps themself, i.e. whether the flaps are already infected or colonized priorly to their reimplantation. However, such studies have been published only sporadically. Chan et al. performed bacterial cultures on 18 explanted skull flaps, which were cryopreserved at − 80 °C for 4–55 months [10]; a positive bacterial growth was observed in 27.8% of the cases. Similarly, Cho et al. examined 47 explanted skull flaps, which were cryopreserved at − 70 °C for 9–161 months and carried out a bacterial culture without bacterial growth [23]. Bhaskar et al. examined 25 explanted skull flaps, which were cryopreserved at − 20 C° for more than 6 months and carried out the bacterial culture with a positive culture rate of 20% [24]. Noteworthy, none of the above studies have analyzed the infection potential of the explanted skull flaps after active contamination with specific microorganisms and of course all skull flaps studied were preserved at the same temperature.

To the best of our knowledge, there has been no analysis to date that compared the microbiological profile and infection potential of skull flaps stored at different temperatures at the same institution. Such a comparison would limit various sources of bias observed in studies conducted at different institutions; i.e. differences in the surgical management (indications and timing of DC and cranioplasty, surgical techniques, performance and surgeons) and in the patterns of storage (steps of material collection and sterilization, temperature and time of storage). In our study, we were able to conduct a bacterial culture of skull flaps explanted and preserved at the same institution with sole difference the store temperature (− 23 °C vs.− 80 °C). No differences of the bacterial growth have been observed. Furthermore, the above-mentioned differently cryopreserved skull flaps and CAD (subgroup C) were additionally compared for their infection potential, as their bone fragments were secondary contaminated in a suspension with specific pathogens; we similarly observed no differences of the growth patterns of pathogens between the subgroups.

In conclusion, our study failed to identify a different infectious behavior of skull flaps stored either at − 23 °C or − 80 °C. Large prospective studies are needed to shed more light on this topic.

## Limitations

1. The retrospective nature of the study and the limited study population does not allow for far reaching conclusions. Larger prospective studies are needed.
2. Skulls flaps of group B were longer preserved than those of group A, however no bacterial growth has been observed in none of the groups.

## Data Availability

The datasets used and/or analysed during the current study are available from the corresponding author on reasonable request.

## Abbreviations

DC: Decompressive hemicraniectomy
*S.aureus*: *Staphylococcus aureus*
*S.epidermidis*: *Staphylococcus epidermidis*
*C.acnes*: *Cutibacterium acnes*
CAD: Computer aided designed
MIQ: Microbiological infectious quality standard
MALDI-TOF MS: Matrix assisted laser desorption/ionisation-time of flight mass spectrometry
TBI: Traumatic brain injury
MCA: Middle cerebral artery

## References

[1] El Ahmadieh TY (2013). Surgical treatment of elevated intracranial pressure: decompressive craniectomy and intracranial pressure monitoring. Neurosurg Clin N Am.

[2] Bender A (2013). Early cranioplasty may improve outcome in neurological patients with decompressive craniectomy. Brain Inj.

[3] Dünisch P (2013). Risk factors of aseptic bone resorption: a study after aut ologous bone flap reinsertion due to decompressive craniotomy. J Neurosurg.

[4] Hutchinson PJ, Hoff JT, Keep RF, Xi G, Hua Y (2006). Decompressive craniectomy in traumatic brain injury the randomized multicenter RESCUEicp study (www.RESCUEicp.com). Acta Neurochir Suppl.

[5] Geurts M (2013). Surgical decompression for space-occupying cerebral infarction: outcomes at 3 years in the randomized HAMLET trial. Stroke.

[6] Vahedi K (2007). Sequential-design, multicenter, randomized, controlled trial of early decompressive craniectomy in malignant middle cerebral artery infarction (DECIMAL Trial). Stroke.

[7] Baldo S, Tacconi L (2010). Effectiveness and safety of subcutaneous abdominal preservation of autologous bone flap after decompressive craniectomy: a prospective pilot study. World Neurosurg.

[8] Shoakazemi A, Flannery T, McConnell RS (2009). Long-term outcome of subcutaneously preserved autologous cranioplasty. Neurosurgery.

[9] Hng D (2015). Delayed cranioplasty: outcomes using frozen autologous bone flaps. Craniomaxillofac Trauma Reconstr.

[10] Chan DYC (2017). Cryostored autologous skull bone for cranioplasty? A study on cranial bone flaps' viability and microbial contamination after deep-frozen storage at – 80 ℃. J Clin Neurosci.

[11] Chang V (2010). Outcomes of cranial repair after craniectomy. J Neurosurg.

[12] Schuss P (2012). Cranioplasty after decompressive craniectomy: the effect of timing on postoperative complications. J Neurotrauma.

[13] Lee L (2013). A retrospective analysis and review of an institution's experience with the complications of cranioplasty. Br J Neurosurg.

[14] Aarabi B (2006). Outcome following decompressive craniectomy for malignant swelling due to severe head injury. J Neurosurg.

[15] Honeybul S, Ho KM (2016). Cranioplasty: morbidity and failure. Br J Neurosurg.

[16] Becker K., Reinhard B, Christian E, Christof E, Anton H, Volkhard AJK, Joachim K, Andreas P, Cord Heinrich S, Ulrich V. Microbiological diagnosis of arthritis and osteomyelitis—Part 1. 2014 80.

[17] Wui SH (2016). The autoclaving of autologous bone is a risk factor for surgical site infection after cranioplasty. World Neurosurg.

[18] Tahir MZ (2013). Safety of untreated autologous cranioplasty after extracorporeal storage at − 26 degree celsius. Br J Neurosurg.

[19] Brommeland T (2015). Cranioplasty complications and risk factors associated with bone flap resorption. Scand J Trauma Resusc Emerg Med.

[20] Cheah PP (2017). Autologous cranioplasty post-operative surgical site infection: does it matter if the bone flaps were stored and handled differently?. Malays J Med Sci.

[21] Schwarz F (2016). Cranioplasty after decompressive craniectomy: is there a rationale for an initial artificial bone-substitute implant? A single-center experience after 631 procedures. J Neurosurg.

[22] Korhonen TK (2018). Quantitative and qualitative analysis of bone flap resorption in patients undergoing cranioplasty after decompressive craniectomy. J Neurosurg.

[23] Cho TG (2017). Osteoblast and bacterial culture from cryopreserved skull flap after craniectomy: laboratory study. J Korean Neurosurg Soc.

[24] Bhaskar IP (2013). Microbial contamination assessment of cryostored autogenous cranial bone flaps: should bone biopsies or swabs be performed?. Acta Neurochir (Wien).

